# Case Report: Home-based Management of Severe COVID-19 with Low-dose Tofacitinib

**DOI:** 10.4269/ajtmh.21-0737

**Published:** 2021-10-04

**Authors:** Ashish Sharma, Mohammad Ali

**Affiliations:** ^1^ Fortis Hospital, Noida, Uttar Pradesh, India;; ^2^ Central Government Health Scheme, Parliament House Annexe, New Delhi, India

## Abstract

Human lives and nations’ economies have been adversely affected worldwide by the COVID-19 pandemic. The hyperinflammatory state associated with the disease may be related to mortality. Systemic glucocorticoid is the first-line therapy for cytokine storm. Various immunomodulatory drugs such as tocilizumab and baricitinib have been used in those not responding to glucocorticoid monotherapy. Amid the peak crisis of COVID-19 in India, there was an extreme paucity of medications, oxygen, and hospital beds. We describe three patients with COVID-19 who received low-dose tofacitinib (an oral Janus kinase inhibitor) in addition to moderate-dose glucocorticoid. These patients were treated at their homes, as the hospitals were short of beds. Rapid reduction in hypoxemia along with gradual resolution of other signs of the disease were observed. The results are reassuring regarding the feasibility of managing of severe COVID-19 outside the hospital setting when healthcare resources are overwhelmed by pandemic-related caseload.

## INTRODUCTION

COVID-19 has taken a huge toll on human lives worldwide. The second wave in India was overwhelming. There were no beds available in hospitals, and home-based care was challenging due to shortages in personnel, equipment, and drugs.

Janus kinases (JAKs) are a group of protein kinases involved in signal transduction at cellular level. JAK 1, 2, and 3 are the subtypes, closely linked to the intracellular domain of various cytokine receptors. JAK is activated when a cytokine binds to its receptor. This results in phosphorylation and activation of signal transducers and activation of transcription (STAT) molecules that induce transcription. JAK inhibitors block this cascade, thereby inhibiting the action of various cytokines.^1^ Cytokine storm is a state of hyperactivation of immune cells resulting in excessive release of cytokines. Deleterious systemic effects are seen that may prove to be fatal. We describe our experience with tofacitinib (a JAK inhibitor) in three patients with cytokine storm associated with COVID-19.

Patients included in the study were seen during the peak crisis of COVID-19 in New Delhi (March–May 2021). Reverse-transcription polymerase chain reaction from nasal and oropharyngeal swabs was positive for severe acute respiratory syndrome coronavirus-2 (SARS-CoV-2). Cytokine storm was confirmed by laboratory parameters (discussed further subsequently). All the patients had severe disease and required hospitalization, which was not possible due to unavailability of beds. Therefore, they were treated at home after obtaining informed consent about the risk involved. Monitoring of vital parameters, collection of blood samples, and administration of medications was done by trained nurses during home visits. Oral tofacitinib 5 mg twice daily was administered for 5 days, followed by 5 mg daily for next 5 days, after inadequate response with moderate-dose glucocorticoid.

## DESCRIPTION OF THE PATIENTS

### Patient 1.

A 58-year-old woman was diagnosed with COVID-19 of 6 days’ duration. Her temperature was 103°F, respiratory rate (RR) 38/minute, and oxygen saturation (SpO_2_) 83%. Blood investigations showed C-reactive protein (CRP) 72 mg/L (< 10) and interleukin (IL)-6 100.2 pg/mL (< 4.4). She had received azithromycin, ivermectin, favipiravir, and acetaminophen before presenting to us. Prednisone 40 mg daily and antibiotics were started, along with oxygen inhalation at her home. Four days later (the 10th day of her illness), CRP was 139 mg/L, D-dimer 1070 ng/mL (< 500), and alanine aminotransferase (ALT) 296 U/L (< 40). She became drowsy, and SpO_2_ dropped to 72%. Obtaining a chest radiograph was not possible. Tofacitinib and injection enoxaparin 40 mg subcutaneously daily were added. After 48 hours, her SpO_2_ was 92% on room air. By sixth day, SpO_2_ reached 96%, and sensorium became normal. Prednisone was tapered-off over 10 days.

### Patient 2.

A 30-year-old obese woman (body mass index 36.2) was symptomatic for the previous 12 days. Her SpO_2_ was 70%, blood pressure 106/66 mm Hg, and RR 36/minute with use of accessary muscles for breathing. Oxygen flow of 8 L/minute raised SpO_2_ to 88%. She was taking prednisone, azithromycin, and vitamin C when she consulted us. Blood investigations revealed D-dimer 890 ng/ml and ALT 727 U/L. Cytokine storm was evident because erythrocyte sedimentation rate (ESR) was 59 mm/hour, CRP 181 mg/L, and IL-6 116 pg/mL. Due to unavailability of hospital beds, she was started on home-based treatment with tofacitinib and injection enoxaparin 60 mg daily along with a broad-spectrum antibiotic; prednisone (60 mg/day) was continued. Seventy-two hours later, her SpO_2_ was 94% on room air. Repeat investigations showed marked improvement (Table 1); prednisone was tapered-off over the next 7 days.

**Table 1:** Parameters of patients before and after initiating tofacitinib

		SpO_2_ (%)	CRP (mg/L)	ESR (mm/hr)	Ferritin (ng/mL)	IL-6 (pg/ml)	D-dimer (ng/mL)	AST (U/L)	ALT (U/L)
Patient 1	Before starting tofacitinib	72	139	62	1,086	100.2	1,090	82	96
	After starting tofacitinib	96 (48 hours)	24	32	302	9.4	370	38	41
Patient 2	Before starting tofacitinib	70	181	59	1,520	116	890	429	727
	After starting tofacitinib	94 (72 hours)	41	36	280	9.9	450	56	91
Patient 3	Before starting tofacitinib	84	152	85	626	29.4	6,421	94	240
	After starting tofacitinib	96 (96 hours)	24	42	313	3.4	1,067	44	131

ALT = alanine aminotransferase; AST = aspartate aminotransferase; CRP = C-reactive protein; ESR = erythrocyte sedimentation rate; SpO_2_ = oxygen saturation; IL-6: interleukin-6.

### Patient 3.

**Figure 1.**
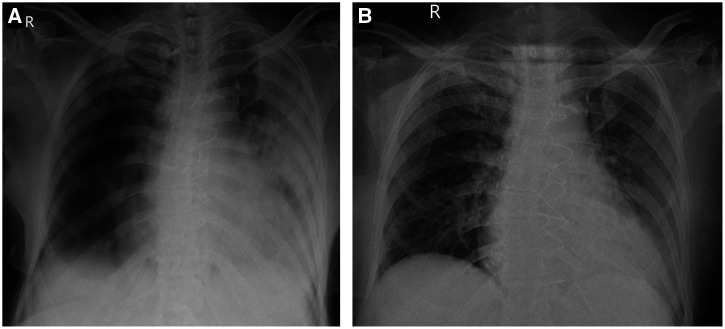
Chest radiographs, posteroanterior projection of Patient 3 on day 1 (**A**) and day 11 (**B**) of presentation.

A 71-year-old woman was seen by us on the 13th day of her illness. Diabetes mellitus type 2, past history of ischemic stroke, and coronary artery bypass grafting for coronary artery disease were the comorbidities. Her SpO_2_ on room air was 84%, improving to 92% at 10 L/minute oxygen flow. Dyspnea was severe and RR was 36/minute. She was being treated with methylprednisolone and azithromycin. Radiograph of the chest showed bilateral infiltrates; left lung was worse (Figure 1A). Blood investigations showed ESR 85 mm/hour, CRP 152 mg/L, ALT 240 U/L, creatinine 1.2 mg/dL (< 0.9), ferritin 626 ng/mL (< 290), IL-6 29.4 pg/mL, and D-dimer 6421 ng/ml. Tofacitinib and apixaban (5 mg/day; enoxaparin was not available due to shortage) were initiated, along with broad-spectrum antibiotic at her home. Methylprednisolone was switched to prednisone 60 mg daily. Ninety-six hours after initiating treatment, patient’s SpO_2_ reached 96% on room air and blood investigations showed marked improvement (Table 1). By 11th day, D-dimer, and liver and kidney function tests had normalized and there was remarkable improvement in chest radiograph (Figure 1B). A slow tapering of prednisone (over 21 days) was done because of a higher starting dose and multiorgan dysfunction.

## DISCUSSION

Cytokine storm is a potentially life-threatening condition resulting from dysregulated release of cytokines from inflammatory cells. Infections and autoimmune diseases are the commonest causes.^2^ Cytokine production is a natural defense mechanism against infections. They give rise to localized inflammation and become undetectable after their function is over. However, sustained activation of immune system, which occurs in cytokine storm, leads to undesirable effects on various organ systems. It may be seen in COVID-19.^3^

In cytokine storm, nonspecific markers of inflammation, like ESR, CRP, ferritin, triglycerides and D-dimer, may be elevated. Circulating levels of IL-6, IL-18, IL-10, IL-1, IL-2, interferon-γ, and tumor necrosis factor-α may be raised. IL-6 in the master cytokine involved in the pathogenesis of hyperinflammatory states arising from various etiologies.^2^^,^^3^ It mediates its effects with the help of membrane-bound and circulating receptors, which result in activation of intracellular inflammatory cascade via JAK-STAT pathway.^2^ Inhibition of IL-6 signalling by tofacitinib has been demonstrated by McInnes et al.^4^ However, IL-6 is only one of the cytokines involved in hyperinflammatory states. Blocking the final common pathway of many cytokines by JAK inhibitors seems to be a more reasonable approach than blocking IL-6 alone by tocilizumab. Lymphocytic inflammation has been demonstrated in the lungs of COVID-19 patients.^5^ Inhibition of action of JAK-dependent cytokines leads to inactivation of lymphocytes. In addition, the number and function of natural killer cells is reduced.^6^

All three patients described here had hypoxemia and transaminitis suggestive of multiorgan dysfunction (Table 1). In addition, Patient 3 had mild renal derangement. Markers of systemic inflammation were elevated including CRP, ferritin, D-dimer, ESR and IL-6 (Table 1). These observations were suggestive of a cytokine storm. Moreover, marked improvement in all the parameters was observed after the addition of a JAK inhibitor to moderate dose glucocorticoid, targeted toward cytokine storm. Baricitinib, an inhibitor of JAK 1 and 2, has been approved for use in severe COVID-19.^7^ Tofacitinib blocks JAK 1 and 3, with some activity against JAK 2. Our patients were seen during the peak crisis of COVID-19 in New Delhi. The use of baricitinib was difficult because of unavailability and prohibitively high cost. Therefore, tofacitinib was used as an alternative agent. There are no published data on the efficacy of tofacitinib in COVID-19 when the patients described in the manuscript were treated. However, an ongoing trial was present under the National Institutes of Health (NCT04469114, subject recruitment completed). The study by Hayek et al. was published while our manuscript was being prepared. It was a retrospective, uncontrolled study that showed the addition of tofacitinib to dexamethasone in hospitalized patients with severe COVID-19 provided survival benefit. Significant reduction in mortality was observed in the tofacitinib group compared with dexamethasone (odds ratio 0.3, *P* = 0.01).^8^ However, our report is distinct because our patients with severe COVID-19 were treated with low-dose tofacitinib at home, without the aid of specialized medical facilities.

**Figure 2.**
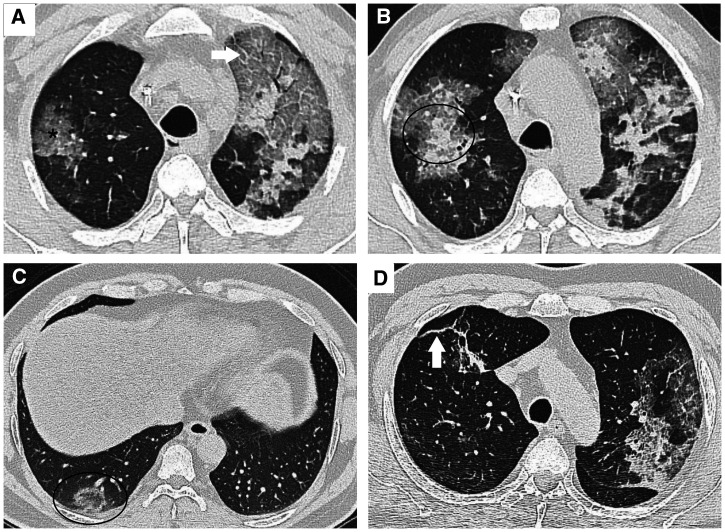
Axial sections of computed tomogram of chest of COVID-19 patients. (**A**) an area of ground-glassing (asterisk) and prominent interlobular septae suggestive of “crazy-paving” pattern (arrow). (**B**) An area of consolidation surrounded by ground-glassing, the halo sign (encircled area). (**C**) An area of ground-glassing surrounded by a rim of consolidation, the reverse halo sign. (**D**) Fibrotic strands (arrow).

In the initial stage of the disease, SARS-CoV-2 colonizes the upper respiratory tract. Some patients progress to develop lower respiratory tract infection. Various features on computed tomography of the chest have been observed in patients with COVID-19 (Figure 2).

Mortality is related to complications of COVID-19 like cytokine storm, pulmonary embolism and septicemia related to secondary infections. Initial stage of viremia and milder disease should be treated with antiviral drugs and acetaminophen. Flare-up of the cytokines is seen after approximately the seventh day of the disease and should be treated with immunosuppressants. Glucocorticoids alone are effective in most patients. However, if used in the initial phase of the disease or in the absence of hypoxemia, more harm than benefit is observed.^9^ Contrary to the results shown by Ramakrishnan et al.,^10^ we have also observed similar detrimental effects with inhaled glucocorticoids in our small cohort, if used early in the disease course (unpublished data). Therefore, correct indication and timing of steroid use is important. As noted earlier, immune activation is an important natural antimicrobial mechanism. Therefore, inhibiting the immune system in the earlier stage may impair clearing of the microbe, thereby leading to worse outcomes.^11^

Dosing of corticosteroids is also important. Six milligram of dexamethasone daily was used in the RECOVERY trial.^9^ A moderate dose of steroids (0.5–0.75 mg/kg prednisone equivalent) is sufficient for most of the patients with cytokine flare. High-dose steroid for prolonged periods, especially in the hospital setting, adds to mortality because it increases the risk of secondary infections. An additional immunosuppressant should be added in those not responding to steroid monotherapy. Drugs targeting specific cytokines such as IL-6, IL-1, and TNF-α have been used with variable success. Nonspecific immunosuppressants such as JAK inhibitors have a wide spectrum of action because they inhibit the activity of many cytokines. Anticytokine drugs and JAK inhibitors are widely available worldwide; however, there are concerns regarding safety and the high cost of therapy. In the setting of an active infection, their use may prove to be disastrous. Most of the targeted immunomodulatory drugs are injectable. On the other hand, JAK inhibitors are oral drugs, which make them more easy to administer.

None of our patients developed secondary infection, herpes zoster, or pulmonary embolism, which are known to occur with tofacitinib.^12^ Patient 3 had significantly raised D-dimer with multiple cardiovascular comorbidities. Treatment with tofacitinib, prednisone, and apixaban led to reduction in D-dimer levels.

Strengths of our report include the rapid tapering of steroids. Moreover, only a moderate dose was used. Low-dose tofacitinib was added only after documenting the failure of moderate-dose steroid monotherapy. In addition, it was used for the shortest period possible, with satisfactory results. However, longer duration may be required in some patients. Prophylactic broad-spectrum antibiotics were used in all of patients because severe sickness is in itself an immunocompromised state. Use of immunosuppressants further increase the risk of secondary infections. As noted earlier, secondary infections contribute significantly to mortality in patients with COVID-19.

Limitations of the study include the small number of patients and the lack of a control group. Further, a chest x-ray with a portable device could only be obtained in Patient 3. However, progress of the patients was closely monitored by timely conduct of blood investigations in their homes. Our work describes the possible response to tofacitinib treatment in severe COVID-19. The findings are reassuring and should trigger new studies with a larger number of subjects; they cannot be used for recommendations in patient care.
